# Unveiling regulatory variants in the blood transcriptome and their association with immunity traits in pigs

**DOI:** 10.3389/fimmu.2025.1582982

**Published:** 2025-06-05

**Authors:** Teodor Jové-Juncà, Daniel Crespo-Piazuelo, Carles Hernández-Banqué, Olga González-Rodríguez, Lingzhao Fang, Raquel Quintanilla, Maria Ballester

**Affiliations:** ^1^ Animal Breeding and Genetics Program, Institute of Agrifood Research and Technology (IRTA), Torre Marimon, Caldes de Montbui, Spain; ^2^ R&D Department, Cuarte S.L., Monzalbarba, Spain; ^3^ Center for Quantitative Genetics and Genomics, Aarhus University, Aarhus, Denmark

**Keywords:** gene expression, eGWAS, swine, immune system, lymphocytes, SNP, RNA-seq

## Abstract

Genome wide association studies (GWAS) have been widely used to investigate the association of genetic markers with complex traits in both humans and livestock species. A particular trait of interest, when studying animal robustness and general immunocompetence, is the transcriptomic profile of blood. To identify genetic variants affecting gene expression in pig blood, we performed expression GWAS (eGWAS) in 255 animals from a commercial Duroc population between 8,499,177 imputed single nucleotide polymorphisms (SNPs) and the expression levels of 14,642 genes obtained from RNA sequencing. Out of the nearly 125 million associations tested, 23 million were found to be significant, grouped in 9,930 expression quantitative trait loci (eQTLs) associated to the expression levels of 6,051 genes. Over 36% of detected eQTLs mapped in close proximity to the genomic location of their associated gene and were classified as *cis-*eQTLs. Moreover, 430,694 variants were found to be associated with the expression of 10 or more different genes and were annotated as transcriptional hotspots. Among genes regulated by these hotspots, we identified genes that encode transcription factors and co-factors regulating immune responses, such as *ARNT*, or co-expressed genes related to immunity (*CSF3R*, *JAK2*, *SOCS3*, *STAT5B* and *UBE2D1*) and associated with health traits, such as phagocytic activity or haptoglobin concentration. In addition, several of the *cis-*regulating variants for immunity candidate genes overlapped with previously described immunity QTLs. Colocalization studies revealed putative common causal variants between the proportion of memory and helper T cells and the candidate genes *CLEC12B*, *IGKV2D*, *KLRC1*, *KLRD1* and *ZAP70*. In conclusion, the associations identified in this study enable the characterization of transcriptional regulators of the pig blood transcriptome. Moreover, the colocalization between immunity QTLs and eQTLs has revealed potential causative mutations regulating immunocompetence in pigs. All these data and results contribute to shedding light on the regulatory mechanisms of blood gene expression and porcine immune regulation.

## Introduction

Genome-wide association studies (GWAS) have been widely applied to study the association between genetic variants and a multitude of phenotypes, including disease incidence in humans or traits related to production performance and animal health in livestock. However, GWAS results showed that the majority of phenotype-associated variants are located in non-coding regions (1), affecting uncharacterized regulatory elements and thus potentially impacting gene expression (1–3). The study of gene expression as an intermediate phenotype is expected to improve the understanding of the relationship between genomic variation and conventional phenotypes (4). Expression Genome Wide Association Studies (eGWAS) are commonly used to find associations between genomic variation and gene expression levels. Genomic regions associated with gene expression are called expression Quantitative Trait Loci (eQTL). If an eQTL is very close to the genomic location of its associated gene, generally less than 1 Mb, it is classified as *cis-*eQTL. Otherwise, they are classified as *trans-*eQTLs (5, 6). Furthermore, genetic variants associated with the expression levels of multiple transcripts, known as transcriptional hotspots, can potentially be key regulators of biological pathways.

In pigs, a number of eGWAS have been carried out using gene-expression microarrays data or a limited set of expressed genes and low-density genotyping arrays (7–12). Nowadays, a combined whole genome and RNA-sequencing (RNA-seq) approach allows much more in-depth analyses (6, 13). Transcriptome datasets from RNA-seq typically encompass about 13,000 genes, which, combined with a vast amount of genotypic data, can result in the identification of thousands of associations between genomic variants and the transcriptome (5). *Cis-*eQTLs have been regularly prioritized over *trans-*eQTLs due to their putative local effect, lower complexity, and larger effect size (4, 5, 14, 15). Traditionally, causal variants affecting gene expression have been searched in upstream regions from the transcription start site (TSS) due to their potential role in promoters or enhancers. However, the potential regulatory effects of *cis-*regulatory variants located in the 3’UTR and *trans*-regulatory elements should not be ruled out (6).

Recently, several studies have improved the understanding of gene expression regulation mechanisms in livestock (6, 13, 16). Initiatives like FarmGTEx (17) or FAANG (Functional Annotation of Animal Genomes) (18) pursue further characterization of gene regulation in farm animals. Peripheral blood transcriptome reflects variations in immune capacity and represents a valuable source of information for identifying biomarkers associated with immunity traits. Characterizing the swine blood transcriptomic profile could enhance our understanding of immune regulation. Furthermore, the functional annotation of the porcine blood transcriptome could facilitate the identification of regulatory markers useful in genomic selection programs aimed at improving sustainability, productivity, and animal health. The characterization of the porcine expression profile could also hold implications for other species, given the pig’s role as a model for studying human diseases and biology. Both species share similarities in physiology, immunity, genome (19, 20), as well as in gene expression profiles (21–23).

In the present work, we aimed to characterize regulatory elements in the pig blood transcriptome and study its association with the expression levels of candidate genes related to immunity traits.

## Materials and methods

### Ethics statement

All procedures involving living animals in this study were performed in compliance with the Spanish Policy of Animal Protection RD 53/2013 under the European Union Directive 2010/63/EU regulating the use of animals in experimentation. All protocols performed were approved by the Ethical Committee of the Institut de Recerca i Tecnologia Agroalimentàries (IRTA).

### Animal material

A commercial Duroc population consisting of 255 healthy piglets (129 females and 126 males) aged 60 ± 8 days was used. Animals belonged to six commercial batches, each containing between 37 and 46 animals. Two to four animals were selected from each litter balancing sex when possible. Animals were raised in the same farm and fed an *ad libitum* cereal-based commercial diet. Blood parameters and *in-situ* physical assessments indicated that the pigs in this study were physically healthy, showing no signs of immunosuppression, subclinical infection, acute stress, or acute/chronic inflammatory responses. Their immunocompetence was within the expected 95% confidence interval (24–26).

Blood was collected via the external jugular vein into vacutainer tubes with anticoagulants (Sangüesa S.A., Spain) and Tempus™ Blood RNA tubes (Thermo Fisher Scientific, Spain) to stabilize the RNA. All samples were transported with ice blocks to the laboratory and stored for further processing at -20°C (for DNA extraction) or -80°C (for RNA extraction).

### Genotyping and imputation

Genomic DNA was extracted from blood samples using NucleoSpin Blood (Macherey-Nagel, Germany). DNA concentration and purity were measured using a Nanodrop ND-1000 spectrophotometer. Genotyping was performed with the GGP Porcine HD Array (Illumina, San Diego, CA) using the Infinium HD Assay Ultra protocol (Illumina). Plink v1.90b3.42 software (27) was used to remove SNPs with a minor allele frequency below 5% and SNPs with more than 10% missing genotypes. SNPs that did not map to the porcine reference genome (Sscrofa11.1 assembly) were also removed. A total of 42,641 SNPs remained for further analysis.

From this dataset, genotype imputation to sequence level was performed using a multi-breed Pig Genomics Reference Panel (PGRPv1) from PigGTEx (13), consisting of 1,602 whole genome sequence samples covering over 100 pig breeds. A total of 42M autosomal biallelic SNPs were imputed using Beagle v5.1 (28). After filtering out variants for dosage R-squared below 0.8 and minor allele frequency below 5%, a total of 8,499,177 SNPs remained for further analysis.

Linkage disequilibrium (LD) analyses were performed with Plink v1.90b3.42 to obtain the number of independent variants in the study. A 0.5 Mb window was used with a cut-off value of r^2^ = 0.7 and a sidestep of 10 SNPs. A total of 118,571 independent variants remained.

### RNA sequencing and mapping

Whole-blood RNA was extracted using Tempus™ Spin RNA Isolation Reagent Kit (Thermo Fisher Scientific, Spain). Total RNA concentration was measured using Nanodrop ND-1000 spectrophotometer. Purity and integrity of RNA was measured using Fragment Analyzer equipment (Agilent Technologies Inc., Santa Clara, CA). All samples had RNA integrity number (RIN) values above 8. Libraries were prepared using the Stranded Total RNA with Ribo-Zero Plus rRNA depletion (Illumina) which removes rRNAs and globin RNAs.

RNA blood samples were sequenced with a depth of >55 M paired end (PE) reads (2 x 150 bp) in an Illumina NovaSeq6000 platform at *Centro Nacional de Análisis Genómico* (CNAG-CRG, Barcelona, Spain). Quality of RNA sequence reads was assessed with the FastQC software (29). RNA sequences were mapped against the reference genome (Sscrofa11.1 assembly) and the Ensembl Genes 109 annotation database using STAR 2.75.3a software (30). Quantification was performed using RSEM 1.3.0 software (31).

Counts were normalized using the EdgeR R package (32) with the trimmed mean of M-values methodology and transformed to counts per million (cpm) using log_2_ with the cpm function. To avoid artefacts, raw counts with a value of 0 were defined as NA. To filter lowly expressed transcripts, a filter similar to the one used by Crespo-Piazuelo et al. (6) was applied. Only genes with cpm above 10/minimum library size in millions (i.e. cpm > 0.69) in more than 35% of the samples were retained. Normality was checked using Shapiro-Wilk test to each expressed gene using a leave-one-out approach. Outliers were removed using this methodology. A total of 14,642 genes remained after filtering.

### Expression genome wide association studies

eGWAS analyses were performed between the normalized expression data and the 8,499,177 SNPs from the imputed data with the fastGWA tool from GCTA 1.93.2 (33), using the following model to estimate the effect of each SNP on the expression level for all expressed genes


$$\text{Y}=\text{X}\beta +\text{Zg}+{\text{S}}_{\text{l}}a_{l}+\text{e}$$

where ***Y*** is the vector containing the expression level for a particular gene of all individuals of the analyzed population; ***β*** stands for the vector of systematic effects sex and batch, being ***X*** the incidence matrix; ***g*** corresponds to the vector of infinitesimal genetic effects of each individual (and ***Z*** the incidence matrix), with distribution $\text{g}\sim N\left(0,\text{G}\sigma^{2g}\right)$ , being ***G*** the genomic relationship matrix calculated as described in Yang et al. (33) and σ^2g^ the additive genetic variance; ***S_l_*** is the vector of genotypes for the *l^th^* SNP, coded as 0, 1, 2, and *a_l_* the SNP allele substitution effect; and finally ***e*** is the vector of residuals.

Multiple testing correction was performed using the Bonferroni method (34). Due to the high linkage disequilibrium detected in the study population, the number of independent tests had to be calculated to avoid overcorrection. As such, the 118,571 independent SNPs were used to compute a significance threshold of p adjust<0.05 (p-value<4.217x10^-7^).

Significantly associated variants were classified as *cis-* or *trans-* in reference to their genomic location respective of the location of their associated gene. Since a drop in LD score was observed at a distance of 1 Mb between variants, SNPs within the 1Mb window from their associated gene were considered to have a local effect on gene expression and were classified as *cis*. Variants outside this window were classified as *trans-*associated variants.

Functional predictions of the significant polymorphisms were performed with the Variant Effect Predictor (VEP) tool (35) on the Ensembl genes 109 annotation database. Significant variants were mapped against the mammalian conserved regions identified by Genomic Evolutionary Rate Profiling from multiple sequence alignments of 103 different mammal species (36).

### Expression quantitative trait loci

eQTL regions were defined by grouping consecutive polymorphisms that were significantly associated with the same gene and located less than 1 Mb apart from each other. To reduce the number of false positives, only eQTLs with a minimum of 3 significantly associated polymorphisms were retained. Then, eQTL regions were extended 0.5Mb on each side in order to take surrounding genetic features in consideration during further analyses. Gene positions were extracted with the Biomart tool (37) from the Ensembl genes 109 database. Similarly to significant variants, eQTL regions were annotated as *cis-*eQTL when significant polymorphisms were located within less than 1Mb from their associated gene, whereas the remaining significant regions were considered as *trans-*eQTL. All genes whose expression was associated with an eQTL were subjected to functional annotation through pathway analysis using the ClueGO Cytoscape plugin (38), in order to identify candidate genes involved in immune-related functions.

### Colocalization studies

In previous studies by Ballester et al. (25, 39) in the same Duroc population, health-related phenotypes were measured from blood, saliva and hair samples extracted at 60 ± 8 days of age. GWAS analyses were performed between the 42,641 SNPs genotyped with the GGP Porcine HD Array (Illumina, San Diego, CA) and 40 health-related traits including immunity traits (25, 39).

As a first approach to identify regulatory regions associated with both immunity phenotypes and gene expression levels, health-related QTLs located on autosomal chromosomes were overlapped with *cis-*eQTLs. Among genes regulated by the overlapping *cis-*eQTLs, candidate genes related to the QTL phenotype were identified using Gene Ontology annotation.

The SNPs associated to both health traits and the expression levels of candidate genes were proposed as putative regulators of immunity. To further assess possible causal variants shared between health QTLs and blood eQTLs, as well as to discriminate between variants within the same *cis-*eQTLs, the coloc-R package version 5 (40) was used. This software uses Bayesian testing and needs to be run between GWAS-like studies with the same variant panel. Therefore, GWAS for the health-related phenotypes were repeated using the imputed 8,499,177 SNPs panel developed in this work. GWAS were performed using a similar model than the one used for eGWAS:


$${\text{Y}}_{\text{p}}=\text{X}\beta +\text{Zg}+{\text{S}}_{\text{l}}a_{l}+e$$

Where ***Y_p_***, was the vector of individual for any health-related trait, and the rest of terms were as described above. The fixed effects considered in ***β*** differed across traits according to those used by Ballester et al. (25, 39).

We used the *coloc.abf* function for the region encompassing both the QTL and eQTL studied. For each pair, four different hypotheses were tested and contrasted with the null hypotheses H0 (no causal SNPs for gene expression nor for the trait), H1 considered causal SNPs only for gene expression, H2 considered causal SNPs only for the health-related trait, H3 considered two different causal SNPs for each trait, and H4 considered a common causal regulator for both. Colocalization was considered to occur when the posterior probability of H4 was greater than 0.95.

Coloc output reported the posterior probability of H4, i.e. having a common regulator for both regions, as well as a list of shared potential causal variants. VEP software was used to map putative common causal variants. SNPs mapped inside regulatory regions (e.g., enhancers and upstream variants) were selected as potential candidates for gene expression regulation. A computer-assisted identification of putative transcription factors binding sites (TFBSs) disrupted by these variants was performed. A genomic sequence of 100 bp surrounding each candidate variant was extracted from Ensembl (pig reference genome Sscrofa11.1). Two sequences for each variant, corresponding to the reference and alternative alleles, were uploaded to the FIMO software from the MEME suite (41) with default parameters, along with a list of motifs corresponding to mammalian TFBSs obtained from the Jaspar database (42). Alignments between TFBSs and sequences containing candidate regulatory mutations with a p-value inferior to 0.001 were selected as significative. TFBSs of transcription factors (TFs) related to immunity, according to Gene Ontology database, were prioritized. The common causal variants affecting TFBSs were selected as candidates for key regulators of immunity through the regulation of gene expression.

### Hotspot detection

Genetic variants associated with the expression of at least 10 genes were considered as genetic hotspots. Furthermore, the most significantly associated genetic variants in at least 10 eQTL regions were defined as top-hotspots (6). Genes with associated hotspots were examined for transcription factors and cofactors using the AnimalTFDB v4.0 (43). Pathways analysis was performed for genes regulated by a single top-hotspot through network analyses with the ClueGO Cytoscape plugin using KEGG pathways and gene ontology databases (38, 44). Co-expression between each regulator and its *trans-*associated genes was assessed using Partial Correlation and Information Theory procedures with the PCIT R package (45).

## Results

### Porcine blood transcriptome

The porcine blood transcriptome sequencing provided an average of 161.5 million reads per sample, totaling over 35.5 billion reads across the entire dataset. Of these, 90.1% were successfully mapped to the pig reference genome Sscrofa11.1. Among these mapped reads, 91.2% were located within genic regions, with 44.06% mapping to exons and 47.09% to introns.

After normalizing and filtering, we identified 14,642 annotated genes as being expressed in blood. The majority of these, 12,461 genes (85.1%), were protein-coding genes, while 2,064 genes (14.1%) were classified as long-non-coding RNAs (lncRNA).

### Genomic variants associated with blood transcriptome

**Figure 1:**
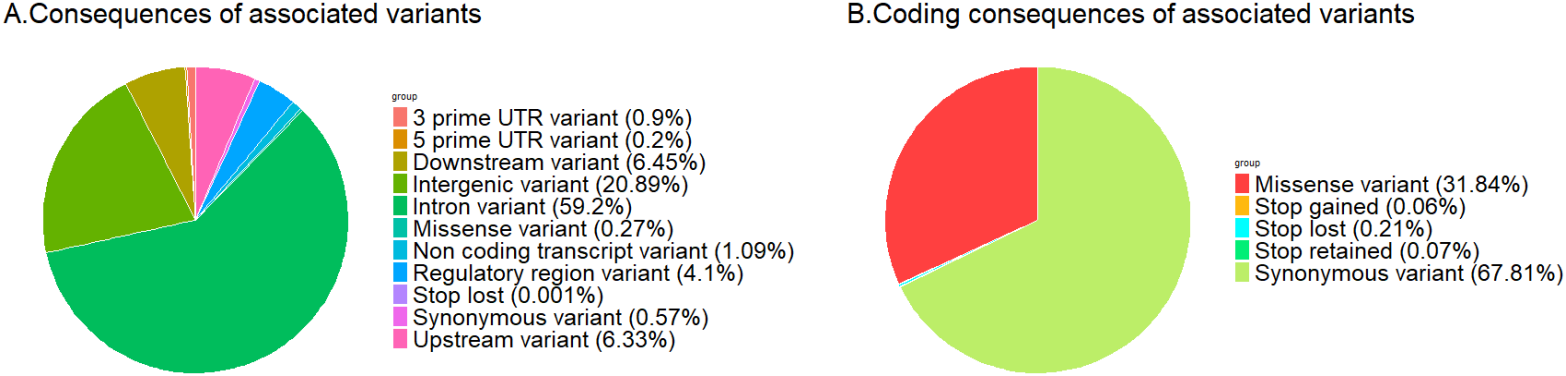
Pie chart presenting the consequences of genetic variants associated to blood gene expression levels **(A)** all associated variants; **(B)** variants in coding regions.

Significant associations between the expression levels of 6,051 genes and 5,682,600 genetic variants were obtained in the eGWAS (**Table 1**). Most of the associated variants were located within intronic (59.2%) and intergenic (20.9%) regions. Only 1% of associated variants involved coding regions, most of which were synonymous variants (67.8%). Regions with a potentially strong regulatory role, such as upstream or UTR, constituted 11.5% of the associated variants (**Figure 1**).

**Table 1:** Summary of results from the blood expression genome wide association study.

Genetic feature	Total	*Cis*	*Trans*
Variants	5,682,600	3,859,132	4,063,000
eQTLs	9,930	3,597	6,333
Hotspots	430,694	344,328	86,366
Top-hotspots	145	42	103
Regulated genes*	6,051	3,952	5,466

*Before filtering by eQTL.

**Figure 2:**
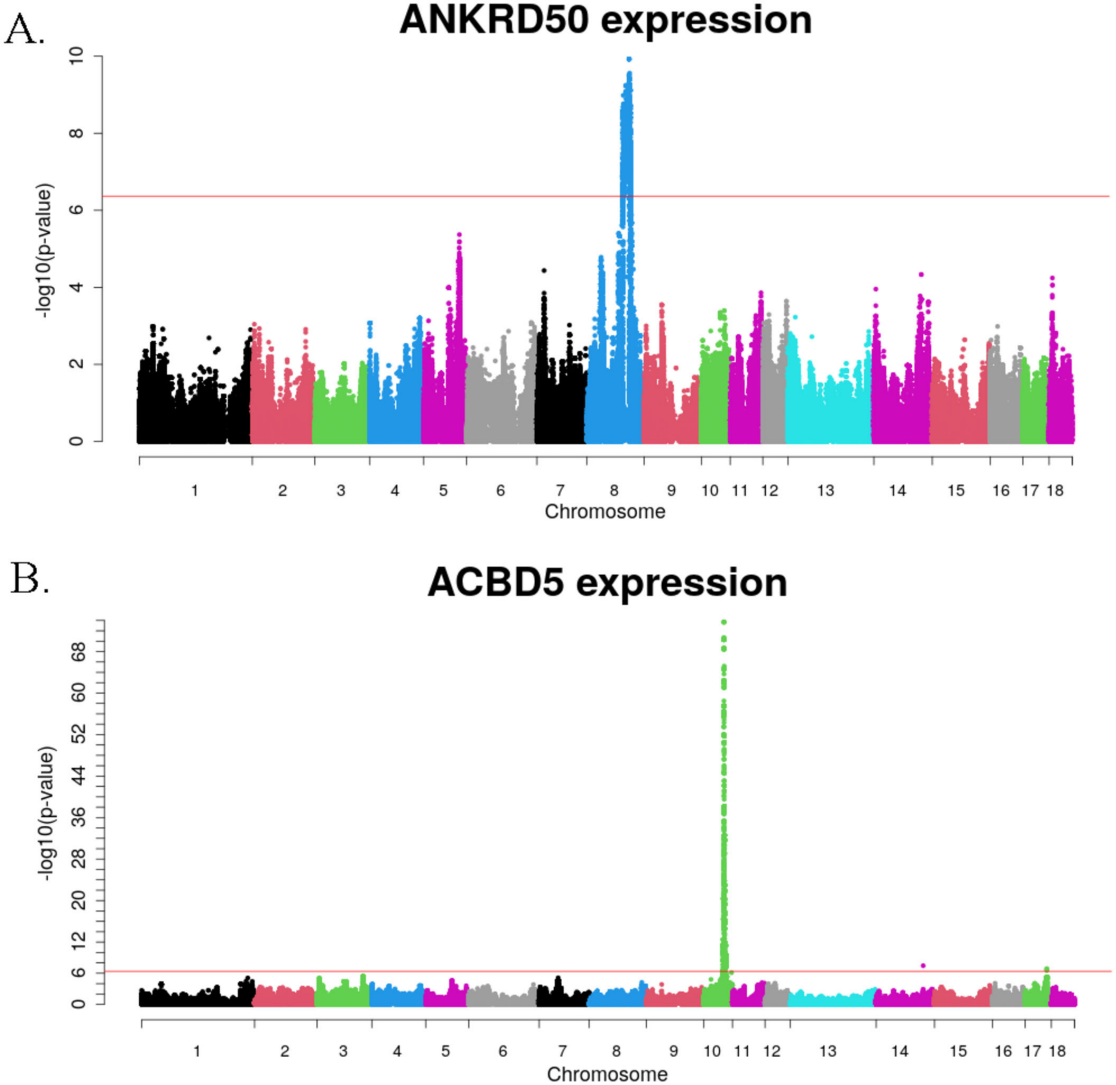
Manhattan plots of eGWAS results for *ANKRD50* **(A)**, and *ACBD5* **(B)** expression levels. The red threshold corresponds to an adjusted p-value of 0.05 after Bonferroni correction for multiple testing.

Of the 6,051 genes associated with genetic polymorphisms, 4,964 were protein-coding, 1,033 were lncRNA, and the remaining were identified as miRNA, snoRNA and other small RNA molecules. The *ANKRD50* gene had the greatest number of associations, with a total of 65,889 genetic variants located on *Sus scrofa* chromosome (SSC) 8 showing a significant effect on *ANKRD50* expression levels (**Figure 2A**). Meanwhile, the expression of *ACBD5* gene exhibited the most significant associations (p-value of 2.02x10^-74^) with the variants *rs335102081*, *rs345896251*, *rs338023300*, *rs697323536* and *rs336561290*, in SSC10 (**Figure 2B**).

When examining individual variants, a total of 3,859,132 (67.9% of significant variants) were found to have at least one association in *cis* (**Table 1**). Focusing on regulated genes, out of the 6,051 genes whose expression levels showed associations at genome level, 3,952 had at least one *cis-*regulatory variant, while 2,099 had exclusively *trans-*regulatory elements. On average, *cis* associations showed lower p-values than *trans* associations.

**Figure 3:**
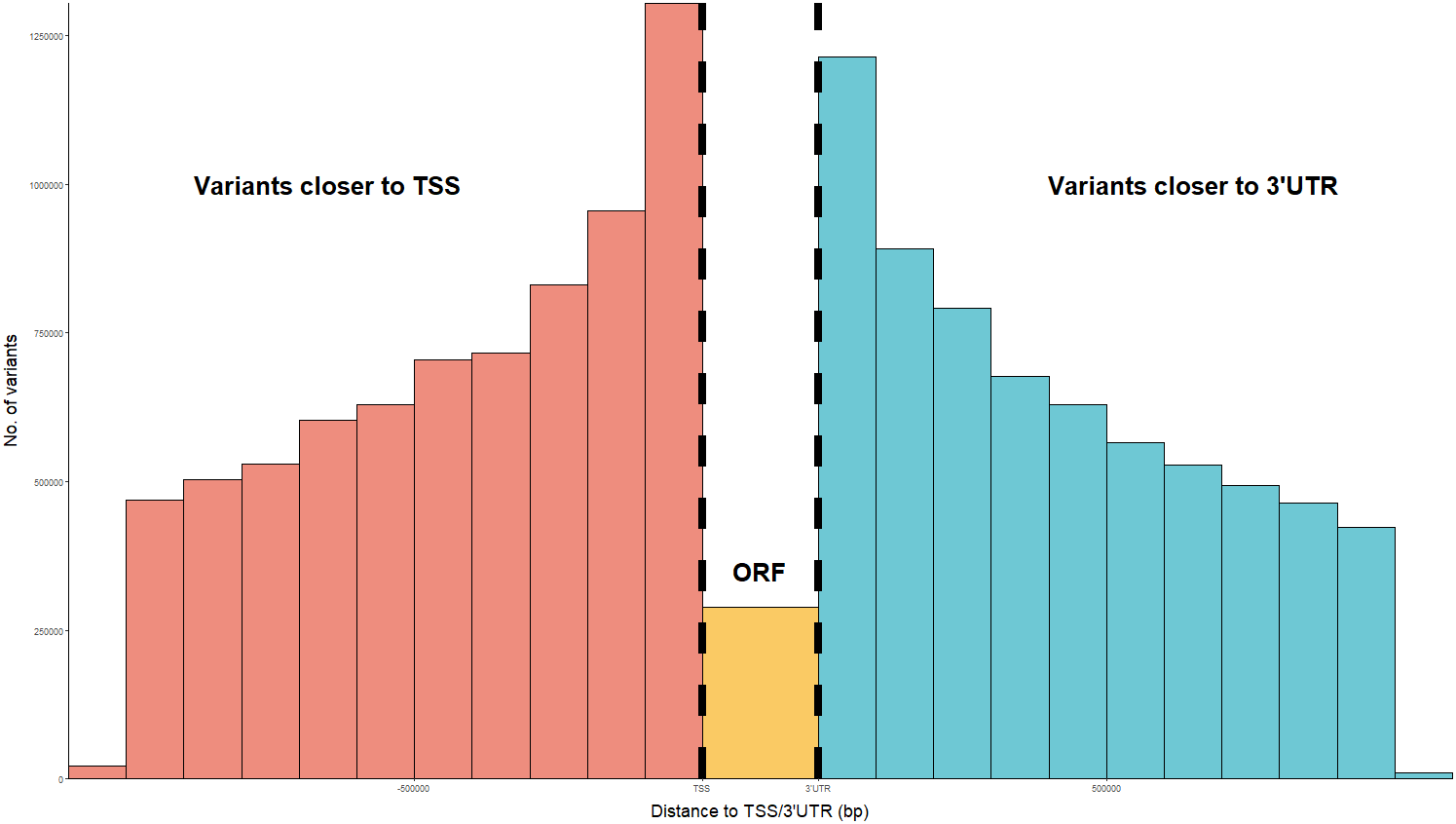
Distribution of the distance between *cis* regulatory variants and their regulated gene ORF, in bp. Distances have been calculated as relative to the transcription start site (TSS) or start of the 3’ untranslated regions (3’UTR), whichever closest. The number of regulatory variants located inside the ORF between the TSS and start of 3’UTR has been plotted in orange.

Variants located in potential regulatory regions were considered as candidates for regulatory elements. According to VEP prediction, a total of 517,770 *cis-*associated variants were found in upstream regions, while 77,350 variants were found in 3’UTR regions. Moreover, 342,114 *cis-*regulatory variants mapped inside regulatory regions. Overall, the percentages of predicted consequences for *cis-*regulatory variants were similar to those observed for all associated variants (**Supplementary Figure 1**). Analysis of the positions of *cis-*associated variants relative to their associated loci revealed an enrichment of associations near the TSS and just beyond the start of the 3’UTR, compared to variants located within the ORF itself (**Figure 3**).

Additionally, 41,781 variants associated with the expression of 4,125 genes mapped inside evolutionarily conserved mammalian regions (GERP regions). The *ANKRD50* gene, involved in endocytic recycling, showed the highest number of associated variants in conserved regions, all inside a single eQTL.

### Genomic regions associated with blood transcriptome

Significant associations found within a distance of ±1 Mb of each other were considered part of the same eQTL. This approach led to the annotation of 14,243 different eQTLs. To ensure robustness and minimize the influence of isolated signals, we focused subsequent analyses on the 9,930 eQTL containing 3 or more significant variants, as the presence of multiple significant variants in close proximity is expected due to LD.

**Figure 4:**
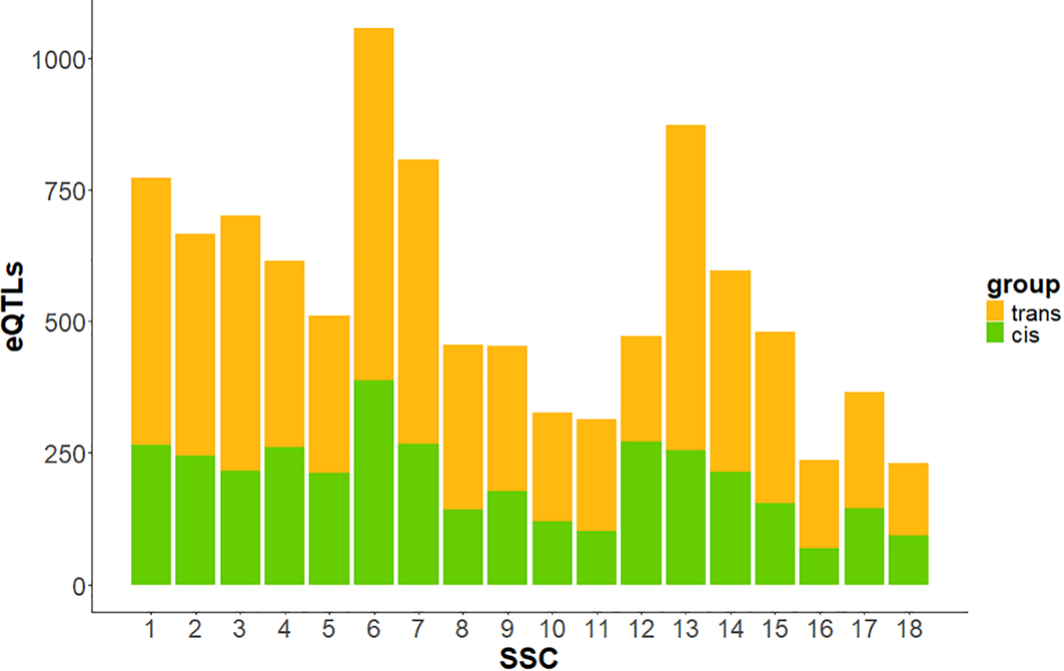
Bar plot showing the number of *cis-* and *trans-*eQTLs across autosomes. In green, eQTLs annotated in *cis-* to their associated gene; in orange, eQTLs annotated in *trans*- to their associated gene.

At eQTL level, 3,597 regions (36.2%) were identified as *cis-*eQTLs, while 6,333 were classified as *trans* (**Table 1**), regulating 3,663 different genes. C*is-*eQTLs comprised a larger number of variants compared to *trans-*eQTLs, with an average of 5,579.52 SNPs per *cis-*eQTLs, whereas *trans-*eQTLs averaged 564.11 SNPs. The genomic distribution of *cis-* and *trans-*eQTLs across the 18 *Sus Scrofa* autosomes is shown in **Figure 4**. SSC6 exhibited the highest number of both total and *cis*-eQTLs, while SSC12 showed the highest proportion of *cis-*eQTLs among all autosomes.

To further characterize immune-related genes regulated by eQTLs, we performed a functional annotation analysis of all eQTL-regulated genes. This analysis identified 771 immune-related genes, 482 of which were regulated in *cis*. The results of the functional annotation are presented in **Supplementary Table S1**.

### Transcriptional hotspots and top-hotspots

To identify key regulatory elements, genetic variants that were significantly associated with the expression of at least 10 genes were classified as transcriptional regulatory hotspots. A total of 430,694 hotspot variants were identified in our study (**Table 1**). The variant with the highest number of associated genes was *rs1107483072*, which regulated the expression of 127 genes. This variant is located inside the sixth intron of the *CAMTA1* gene, a transcription factor related to several immunological pathways (46).

**Figure 5:**
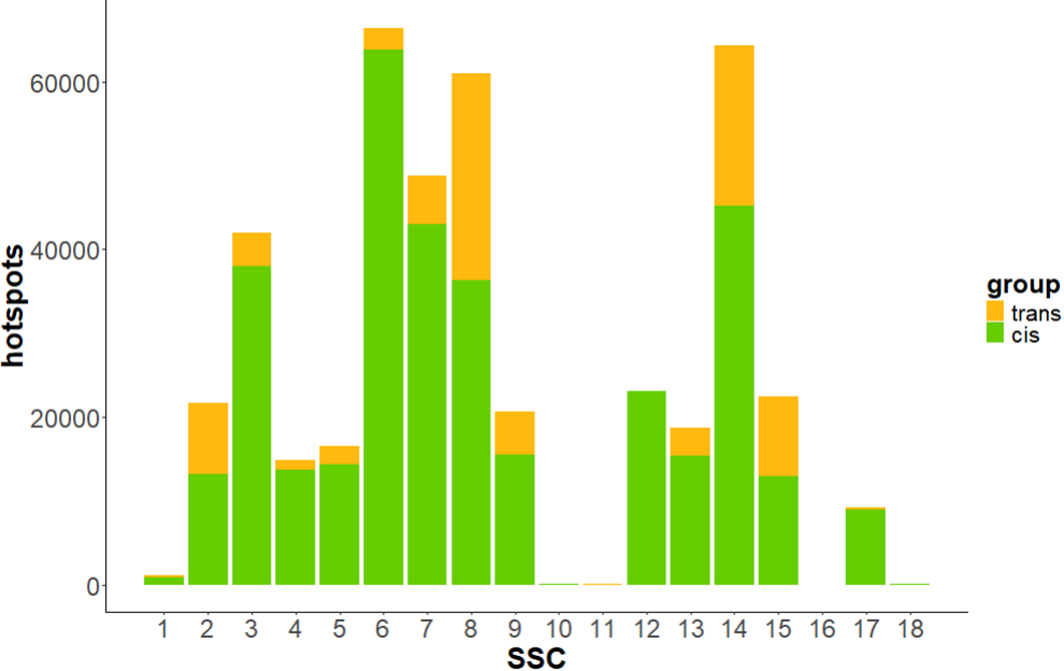
Bar plot depicting the distribution of *cis-* and *trans-*hotspots across autosomes. In green, hotspots annotated in *cis* to at least one of its associated genes; in orange hotspots annotated in *trans* to all its associated genes. Chromosomes SSC10, SSC11 and SSC18, with less than 150 hotspots, were not plotted due to the relative bar size.

Hotspot distribution across the genome was not uniform. The chromosomes with the highest number of hotspots were SSC6, SSC14 and SSC8, comprising 66,351, 64,393 and 61,022 hotspot variants, respectively (**Figure 5**). While most of these variants appeared functionally neutral, a subset of 422 was predicted to have deleterious effects on the protein sequence, and an additional 363 variants were also predicted to be deleterious but with low confidence. Among variants classified as hotspots, 344,328 (79.9%) were found in *cis* with at least one of their associated genes.

The 2,093 genes associated with transcriptional hotspots included a significant number of transcriptional regulators: 112 were annotated as transcription factors and 106 as transcription co-factors. Of these, 68 transcription factors and 49 co-factors were found to have *cis-*hotspot associations. Approximately half of the hotspots associated in *cis* with a transcription factor or co-factor (46.9% and 46.2%, respectively) mapped inside intronic regions. Additionally, 13,462 hotspots in *cis* with transcription factors and 10,047 hotspots in cis with transcription co-factors mapped in regulatory regions. When looking at 3’UTR regions, 2,251 *cis-*hotspots associated with transcription factors and 1,719 *cis-*hotspots associated with transcription co-factors were mapped inside.

Among the total hotspot variants identified, 144 were also the most significantly associated polymorphisms in at least ten eQTLs. These were considered transcriptional top-hotspots and are detailed in **Supplementary Table S2**. All top hotspots grouped in seven genomics regions that are described in **Table 2**. From them, 42 variants were found to be associated in *cis* with at least one transcript in which they were the top association of the *cis-*eQTL. Functional annotation of genes regulated by the same regulatory top-hotspot highlighted biological processes regulated by the same regulatory variants. Results from ClueGO pathway analyses and PCIT co-expression analyses can be found in **Supplementary Table S3**.

**Table 2:** Top-hotspots detected during the analysis, grouped by linkage disequilibrium.

SSC	Start (Mb)	End (Mb)	n° of variants	n° of *cis-*associated variants	n° associated genes	n° top associated genes	n° *cis-* associated genes	*cis-*associated genes
1	262.86	262.86	1	0	23	10	0	NA
11	24.22	24.22	1	0	11	11	0	NA
12	61.56	61.56	4	4	18	10	8	*ATPAF2*, *DRC3*, *ENSSSCG00000055301*, *NATD1*, *NT5M*, *SREBF1*, *TOM1L2*, *USP22*
15	100.94	100.94	1	1	20	10	1	*HECW2*
2	39.05	39.05	11	0	60	20	0	NA
6	68.24	68.32	37	37	154	53	1	VAMP3*
8	120.00	120.04	89	0	30	13	0	NA

SSC stands for *Sus scrofa* chromosome number. * Signifies that the gene was detected by homology with human.

**Figure 6:**
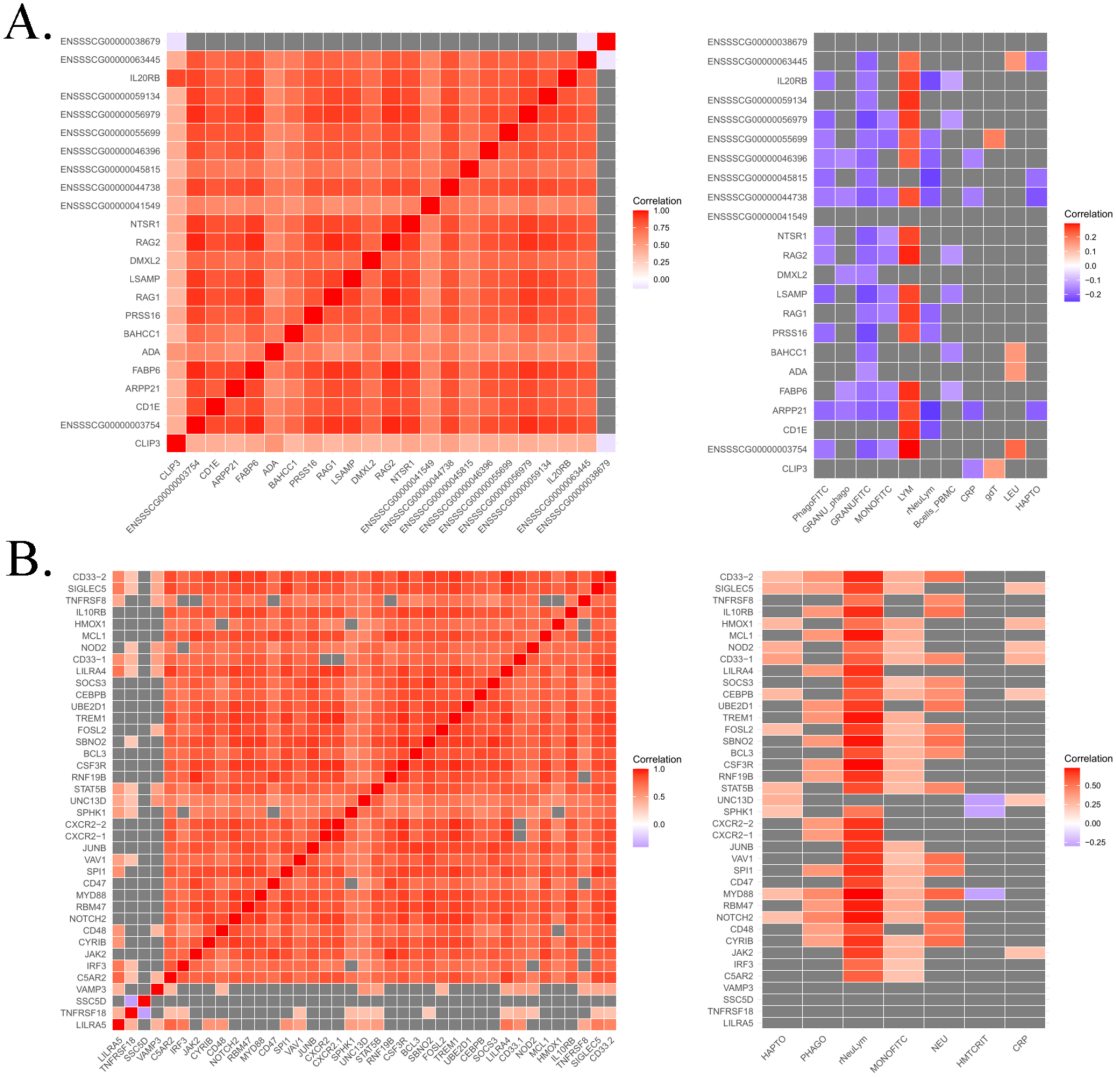
Heatmap of partial correlations from PCIT analysis between the expression levels of genes regulated by top-hotspots in SSC1 **(A)** and SSC6 **(B)**, as well as their correlations with immunity traits. Positive correlations are presented in red, negative ones in blue, and non-significant correlations in grey.

For instance, a top-hotspot was identified on SSC1 associated with the expression of 23 genes. Co-expression analysis revealed strong correlations ranging from 0.56 to 0.95 between the expression levels of these genes, except for the novel gene *ENSSSCG00000038679*, which encodes a centrosomal protein. The functional enrichment analysis revealed several genes related to immune functions, including those involved in the T-cell apoptotic process (*ADA*, and *RAG1*). Other genes such as *RAG1* and *RAG2*, both involved in activation of V(D)J recombination, were also identified. The PCIT analysis reported significant and negative correlations of the expression levels of most of these genes with phagocytosis, whereas positive correlations were observed with lymphocyte count (**Figure 6A**).

A total of 37 top-hotspots in high LD were located in SSC6, all of them associated in *cis* to the expression levels of *VAMP3* gene and located in intron 6 of the transcription factor *CAMTA1*. After VEP annotation, one of these top-hotspots, the *rs3472545010*, was within an enhancer (*ENSSSCR6_9324TC*). Remarkably, these top-hotspots were found to regulate genes associated with biological processes and pathways related to immunity such as signaling by CSF3 (G-CSF), regulation of interleukin 1, 8 and 10 production, regulation of tumor necrosis factor production or interferon-mediated signaling pathway among others (**Supplementary Table S4**). Furthermore, *VAMP3*, together with *MYD88*, *SBNO2*, *SPI1*, and *UNC13*, were found to be associated to the myeloid cell activation involved in immune response biological process. Co-expression analyses revealed strong correlations between the expression levels of these genes putatively co-regulated by the same top-hotspots (**Figure 6B**). Furthermore, significant correlations were observed between the expression of genes regulated by these hotspots and several health-related traits such as plasma concentration of acute phase proteins (Haptoglobin and CRP), phagocytic cells and phagocytic capacity of monocytes, neutrophil count, and the neutrophil/lymphocyte ratio (**Figure 6B**).

Four top-hotspots in high LD located on SSC12 presented *cis-*associations with eight genes: *ATPAF2*, *DRC3*, *ENSSSCG00000055301* encoding a lncRNA, *NATD1*, *NT5M*, *SREBF1*, *TOM1L2* and *USP22*. Notably, one of these putatively regulated genes encodes the transcription factor *SREBF1* that plays a key role in steroid metabolism and lipid homeostasis. The only other *cis-*association found for a top-hotspot was between the variant *rs3474314913* located on SSC15 and the gene *HECW2*.

### Potential causal regulatory variants for immunity traits

The comparison of the blood transcriptome eQTL map with QTLs of health-related traits reported a total of 294 eQTLs, 175 of which were *cis-*eQTLs, colocalized with 15 hematological and immunity QTLs. Results are shown in **Table 3**. The immunity QTL with the highest number of colocalizations was the QTL at SSC3 for the relative abundance of T-helper cells among PBMCs, which had 45 overlapping eQTLs, and 17 of which were in *cis*. Next, the QTL at SSC5 for memory T cell amongst PBMCs had 41 overlapping eQTLs, and 22 of them in *cis*. Other QTL of phenotypes such as IgG serum or CRP concentration, white blood cells and platelets counts, mean corpuscular volume (MCV) and mean corpuscular hemoglobin (MCH) also colocalized eQTLs for blood transcriptome. The gene ontology enrichment analysis of genes regulated by these colocalized eQTLs revealed several candidate genes (**Table 3**) associated with immune responses. Among them, *CD48* and *SLAMF* family receptors genes overlapped with CRP concentration in serum, *IGHG4* and *IGHV3–73* with IgG plasma levels and *PIK3R3*, *PRDX1* and *MPL* with MCV and MCH. Several members of the *IGKV*, *RBPJ*, and *ZAP70* genes, were also identified as candidate genes for the abundance of T-helper cells, while several members of the *CLEC* and *KLR* gene families were for memory T cells.

**Table 3:** Number of overlapping eQTLs to previously described immune associated regions.

Immunity Trait	Identified QTL			Overlapping eQTL		Candidate genes
	SSC	start (Mb)	end (Mb)	Number of eQTL	Number of *cis*-eQTL	
Platelets count (n/μL)	16	21.98	23.22	12	7	
CRP in serum (μg/ml)	4	90.54	90.8	25	18	CD48, SLAMF1/6/8/9, ATF6, S100A13*, FCER1A
Granulocytes phagocytosis FITC	12	8.57	8.62	15	14	CD300A*, ABCA5
Granulocytes phagocytosis (%)	5	69.13	70.23	18	6	
Mean corpuscular hemoglobin (pg)	6	164.85	165.85	27	19	*PIK3R3, PRDX1, MPL*
IgG in plasma (mg/ml)	4	8.38	8.87	7	3	
IgG in plasma (mg/ml)	7	117.1	117.29	8	4	IGHG4*, IGHV3-73*
Leukocytes count (n/μL)	14	123.53	124.29	6	5	
Lymphocytes count (n/μL)	17	52.47	52.51	6	4	
Neutrophils count (n/μL)	13	69.03	71.96	31	20	*MAGI1*
Mean corpuscular volume (fL)	6	84.96	84.98	22	13	*CASP9*
Mean corpuscular volume (fL)	6	164.85	165.91	27	19	*PIK3R3, PRDX1, MPL*
T-helper cells (%PBMC)	8	20.52	20.58	4	4	*RBPJ*
T-helper cells (%PBMC)	3	55.6	58.34	45	17	IGKV@, ZAP70*
Memory T-helper cells (%PBMC)	5	61.62	62.44	41	22	KLRD1, KLRC*, CLEC/4/7/12, PHC1
Total				294	175	

SSC stands for *Sus scrofa* chromosome number.

*Detected by homology.

Colocalization studies revealed substantial evidence of common causal regulators between health-related QTLs and the eQTLs described in the present work; **Table 4** shows results for tests reporting posterior probabilities of having common causal variants above 0.95, plus the candidates to be the key regulators. The QTL for memory T cell showed common causal mutations with three *cis-*eQTLs for *CLEC12B* (PP.H4 = 0.99), *KLRD1* (PP.H4 = 0.97) and *KLRC1* (PP.H4 = 0.96) gene expression. The QTL for T-helper cell percentage also presented putative common causal variants with eQTL for several homologues to the *IGKV2D* gene (PP.H4 from 0.96 to 0.99) and *ZAP70* (PP.H4 = 0.96).

**Table 4:** Colocalization analysis results.

Gene	eQTL SSC	eQTL start	eQTL end	Trait	QTL SSC	QTL start	QTL end	PP.H4	Candidate variants	Candidate TFBSs affected by candidate variants
*CLEC12B*	5	30.8	68.81	Memory T cells %PBMC	5	62	62	99.05	*rs327287009, rs321672514, rs320481119, rs1113891078, rs1113630513*, *rs332711503, rs691734190, rs324109474, rs329194838*, *rs324908876*, *rs325546963*	CEBPG, CREB3, ELF1, ELF2, ELF4, FLI1, FOXP3, GABPA, IRF2, IRF3, IRF4, IRF7, IRF9, JUN, RORA, ZBTB7A
*KLRD1*	5	61.4	62.8					97.84		
*ENSSSCG00000036743*	5	61.4	64.47					96.81		
*ENSSSCG00000040986*	3	39.8	70.93	T-helper cells % PBMC	3	56	58	98.61	*rs340907534, rs707038723, rs329506333, rs81498129, rs330982811, rs340972582, rs320339342, rs337569046, rs340213966, rs318655915, rs334765227, rs319237927, rs323957461, rs328915079, rs324370375, rs335067319, rs332294508, rs341361092, rs345903117*	BATF3, CEBPG, CREB3, CREB3L4, EGR3, ELF4, EOMES, ETS1, FOS, FOXC1, FOXL1, FOXP1, GATA2, GATA6, GLI3, HIF1A, IRF3, IRF8, JUN, KLF10, KLF13, KLF2, KLF4, MEF2C, MITF, MYB, MYC, NKX2-3, NR1D1, PPARG, RARA, RORA, RORC, RUNX3, SOX13, SOX4, SP3, TBX21, TCF3, TFE3, XBP1, ZBTB7A, ZBTB7B, ZEB1, ZNF16, ZNF675
*ENSSSCG00000033114*	3	20.4	75.98					97.85		
*ENSSSCG00000040009*	3	51.4	58.52					97.84		
*ENSSSCG00000054922*	3	29.1	73.51					97.77		
*ENSSSCG00000061405*	3	54	67.63					96.57		
*ZAP70*	3	54	67.63					95.52		

SSC stands for *Sus scrofa* chromosome number, PP.H4 stands for the posterior probability of hypothesis 4.

The 95% credible set of causal variants for the coloc studies was extracted for further analysis. Variants located in regulatory regions were selected as candidate regulators for these QTLs and eQTLs (**Table 4**). Eleven variants were selected for the memory T-cells QTL and *CLEC12B*, *KLRD1* and *KLRC1* eQTLs. An in-silico characterization of potentially modified TFBSs by these variants identified three of them as modifying immune-related TFBSs for CEBPG, ELF1, ELF2 and ELF4, IRF2–4 and IRF7, and RORA, among others (**Supplementary Table S4**). Nineteen variants were identified as potentially shared causal variants among the QTL for Helper T cells and the eQTLs for *IGKV2D* and *ZAP70*. A total of 46 in-silico predicted TFBSs for TFs associated with immune processes, including CEBPG, CREB3, GATA2 and GATA6, RORA, RORC, SOX13, and ZAN16, among others, were identified as being modified by these variants (**Supplementary Table S4**).

### Comparative blood transcriptome with other transcriptomic analyses

**Figure 7:**
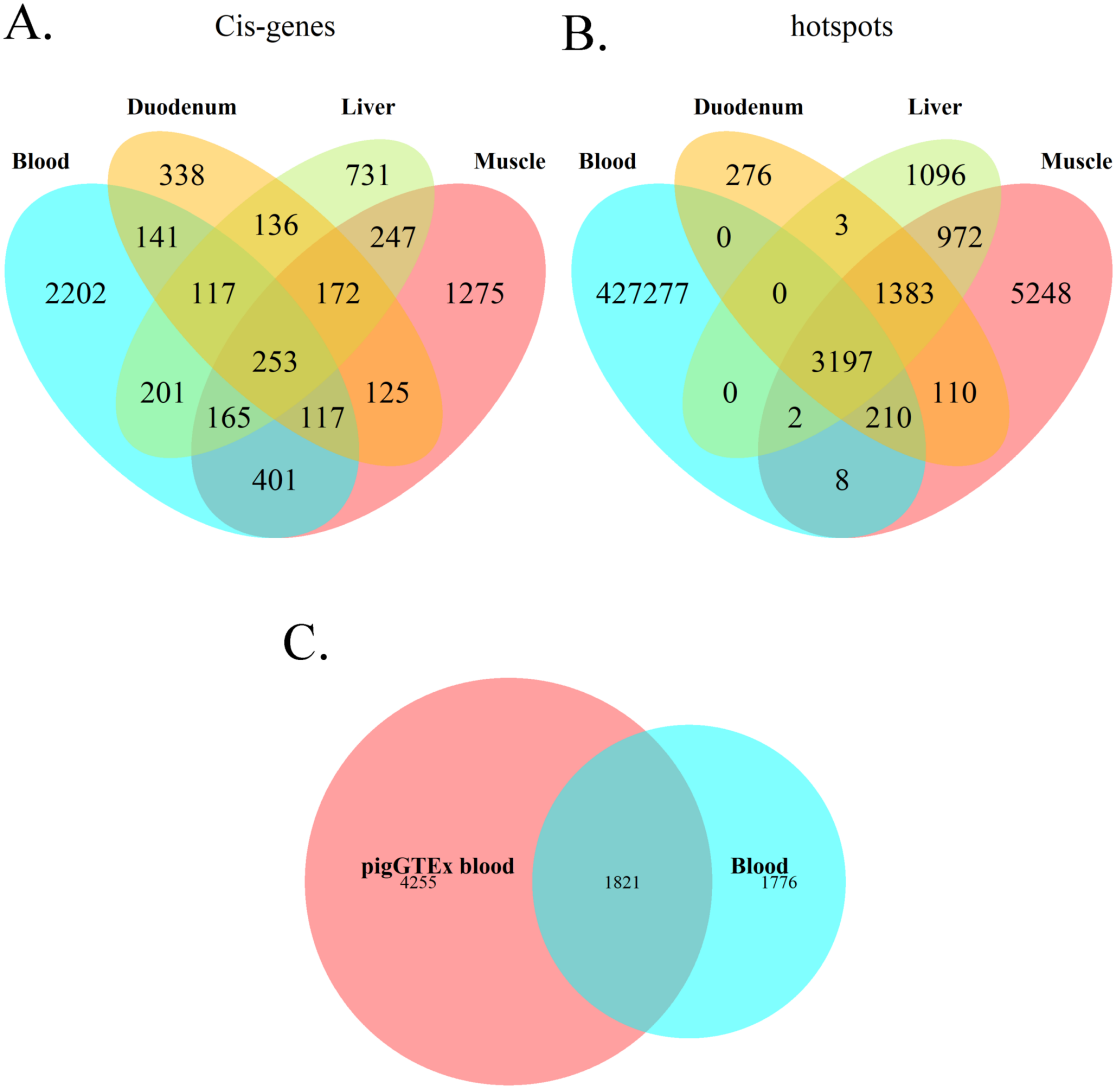
Venn diagrams depicting the overlap of results regarding porcine blood transcriptome with other studies. **(A)** Overlap between genes regulated by a *cis* eQTL in blood and those in duodenum, muscle and liver from Crespo-Piazuelo et al. (6) and the present work. **(B)** Overlap between regulatory hotspots for blood transcriptome and those for duodenum, muscle and liver from Crespo-Piazuelo et al. (6). **(C)** Overlap between genes regulated in blood by a *cis* eQTL in the present study and the PigGTEx project (13).

Genes regulated by *cis-*eQTLs in the blood transcriptome were compared with those identified in previous studies in different porcine tissues. Crespo-Piazuelo et al. (6) analyzed the transcriptome of duodenum, liver and muscle from 300 pigs of Landrace, Large White and Duroc breeds. The duodenum transcriptome presented the highest proportion (44.89%) of *cis-*regulated genes overlapping with those identified as *cis-*regulated in blood, followed by liver and muscle (36.40% and 33.97%, respectively) (**Figure 7A**). When comparing results from all tissues, a total of 253 *cis-*regulated genes overlapped in all four tissues. Of these genes commonly regulated across tissues, 17 were annotated as transcription factors, and 12 as transcription co-factors, while the remaining genes had mostly housekeeping roles (**Supplementary Table S5**). Focusing on hotspot variants, when comparing hotspots in blood transcriptome with those obtained by Crespo-Piazuelo et al. (6), a total of 3,197 variants were identified as transcriptional regulatory hotspots across all four tissues (**Figure 7B**). However, none of them was found to be in *cis* in all four tissues simultaneously.

Additionally, results were compared with blood transcriptome data from the FarmGTEx consortium (13). In this case, 1,821 *cis-*regulated genes were shared between studies (**Figure 7C**). The coincidences can be found in **Supplementary Table S5**.

## Discussion

The present study focuses on the genetic regulation of the porcine blood transcriptome, aiming to identify transcriptional regulatory variants and disentangle their association with immune capacity. RNA-seq data from 255 Duroc pigs were used to investigate the association between 8M imputed polymorphisms and expression levels of 14,642 genes in blood. A total of 23,645,971 significant associations, grouped into 9,930 eQTLs linked to the expression levels of 6,051 genes, were reported.

Among these significant eQTLs, 36.2% were associated in *cis* with the expression levels of 3,952 genes, which corresponded to 65.3% of regulated genes showing at least one *cis-*associated region. This represents a proportion of *cis-*eQTL similar to that observed in other studies (e.g. Crespo-Piazuelo et al., and Farhangi et al.) (6, 47). Moreover, *cis-*associated regions tend to be larger in both the number of variants and significance level than *trans-*eQTL regions, as was also observed by Dixon et al. (48) and Lim et al. (49). The *cis-*regulatory variants were enriched in previously mapped regulatory regions, in line with what had been reported in previous studies (e.g. Dixon et al., Crespo-Piazuelo et al.) (6, 48). Both promoters and 3’UTR regions were similarly enriched by *cis-*regulatory elements, highlighting the importance of considering the regulatory impact of 3’UTR regions (50).

It is worth highlighting those genes expressed in the blood transcriptome showing the most relevant *cis-*associations with genome. The most significant association (p-value= 2.02x10^-74^) was found in *cis* between the expression levels of the *ACBD5* gene and an intergenic region upstream the gene. Currently, no enhancer regions are annotated in this region. However, transcription factor binding sites for GATA1, NFACTC2 and TBP were found inside, which would indicate a possible regulatory role. The gene *ACBD5* is known to affect lipid metabolism through peroxisomal defects (51). However, mutations in *ACBD5* have been seen associated to leukodystrophy and pexophagy, having a possible effect on platelet formation and megakaryocyte differentiation (52, 53). The *NOX4* gene showed the second most significant eQTL (p-value= 2.98x10^-73^), being its expression levels associated with the intergenic SNPs *rs328730607* and *rs331477910*, located upstream of the TSS of the gene. *NOX4* is involved with non-phagocytic cells reactive oxygen species production (54). Thus, is considered a tumor-suppressor gene whose overexpression has been described to impact the efficiency of the transcription factor YY1, macrophage infiltration, and inflammatory capacity (55, 56).

Despite the high incidence of *cis-*eQTL regulating the blood transcriptome, 60.6% of genes putatively regulated at genetic level presented at least one form of *trans-*regulation. The vast majority of these *trans-*eQTLs were found to regulate multiple genes. In this study, we focused on those variants associated to the expression of ten or more transcripts. A total of 430,694 variants were detected as transcriptional hotspots, 344,328 of them *cis-*regulating the expression levels of at least one of their associated genes. Remarkably, SSC6 was among the pig chromosomes with the highest number of hotspots. Similarly, Crespo-Piazuelo et al. (6) reported 102 hotspots located at 56.3-64.5 Mb on SSC6 that were consistently associated to gene expression levels in duodenum, muscle and liver pig tissues, and were predicted to have a moderate or high impact on protein sequences. A total of 77 hotspots on SSC6 associated to blood transcriptome were shared with these hotspots identified in all tissues, supporting the existence of common regulatory elements.

Among the 2,093 genes regulated by the hotspots, 228 were annotated as transcription factors (TF) and co-factors, including several immunity-related TF and co-factors. Among them, the ARNT, a TF involved in the regulation of different biological functions including innate and adaptative immune responses (57). In our study, 2,013 *cis-*hotspots were associated to *ARNT* gene expression. In addition, a missense variant (*rs338823350*) associated with the expression of 10 genes was found on the coding region of the *ARNT* gene. Other co-factors regulating immunity functions and with missense hotspot variants were NLRP12, SIRT2 and KMT5C.

Special attention must be given to the identified top-hotspots, some of which contained top *cis-*regulatory variants associated with at least one transcript. Our results describe top-hotspots regulating co-expressed genes related to immune functions and associated with health traits, making them strong candidates for immunomodulation in pigs. It is worth noting the 37 highly linked top-hotspots found in intron 6 of the transcription factor CAMTA1. These 37 variants, among which *rs3472545010* was located within an enhancer, regulated a total of 154 different genes, with a unique *cis-*hotspot for *VAPM3* gene. The gene *VAMP3* is involved in vesicle membrane adhesion across multiple cellular populations; a particular relevant function of *VAMP3* in blood relies in fibronectin reabsorption and release in platelets via toll-like receptor signaling (58, 59). Among genes regulated by the SSC6 top-hotspots, *CSF3R*, *JAK2*, *SOCS3*, *STAT5B* and *UBE2D1* are implicated in the signaling by the CSF3 pathway. CSF3, also known as G-CSF, is a cytokine that regulates production of neutrophils (60). Remarkably, these genes were highly co-expressed between them and also showed significant correlations with neutrophil counts and the neutrophil/lymphocyte ratio. Another top-hotspot, composed exclusively by the intergenic variant *rs326486411*, showed potential immunological implications. This variant was found *trans*-associated with several immunity candidate genes such as *ADA*, *CD1E*, *IL20RB*, *RAG1* and *RAG2*. The *RAG1* and *RAG2* genes are well characterized immunity markers, being involved in V(D)J recombination (61). Curiously, the expression of several genes regulated by this hotspot correlated negatively with phagocytosis-related traits. Among the candidate genes for immunity mentioned, only *CD1E* and *IL20RB* could be *a priori* tangentially related to phagocytic capacity, given the *CD1E* regulatory functions of dendritic cells (62), and in the case of *IL20RB* involvement pathogens elimination (63). Due to the abundance of genes regulated by *rs326486411* and their relationship with immunity, its intergenic location is a strong candidate for being a regulatory element in the pig genome.

Additional candidate regulators of gene expression with the potential to determine health traits were identified in the present study. The overlapping and colocalization studies with previously QTLs for health traits (25, 39) reported strong candidate genes such as *CLEC12B*, *KLRD1* or *ZAP70* which could influence the percentages of T-helper and memory T-helper cells. Several of the putative causal mutations identified by coloc were located in regulatory regions and affected transcription factor binding sites. Genes sharing putative causal variants with memory T cells were *CLEC12B, KLRD1* and the homologue of *KLRC1*, *ENSSSCG00000036743*, both members of the killer cell lectin-like receptor family. Members of both gene families are highly expressed in memory T cells and have been involved in the activation (CLECs) and killing functions (KLRs) of memory T cells (64). The variant *rs1113630513*, located in an enhancer region (ENSSSCR5_BG8XW), was associated with the expression of all three genes and memory T-cell percentage and was found to affect binding sites for several members of the IRF transcription factor family. Members of the IRF family have been reported to be involved in CD4-positive or CD8-positive, alpha-beta T cell lineage commitment, especially in Helper T cell commitment through interferon I (65). For helper T cells, several members of the *IGKV* gene family and *ZAP70* shared putative common causal variants. The *IGKV* loci in pigs contains several pseudogenes; however, the *IGKV2* variants detected in this study are the most active (66). *ZAP70* is a known regulator of adaptative immunity through T cell development (67). Five candidate putative mutations (*rs707038723*, *rs81498129*, *rs340972582*, *rs337569046* and *rs318655915*), mapped inside enhancers, were found to affect binding sites for RORA, which is known to be involved in T-helper cell differentiation (68). The putative causal mutations identified in this study could impact pig immunity through the regulation of gene expression and could serve as target candidates for enhancing cellular immune responses in pigs. Overall, our results point towards regulators of pig immunity that could be used as biomarkers. Further studies are needed to validate these results and to assess the applicability of these markers in breeding programs.

Finally, the identified regulators of blood transcriptome showed important coincidences with those reported by the FarmGTEx consortium (13) across various experimental setups, reinforcing the robustness of the transcriptomic analysis and highlighting important putative regulators. Furthermore, the comparison of blood transcriptome eQTLs with those observed in muscle, duodenum and liver transcriptomes (6) revealed a high proportion of genes commonly regulated across these tissues, as well as shared regulatory hotspots variants. These results confirm the existence of common transcriptional regulatory mechanisms across tissues, which has already been postulated in previous studies (6, 69–71).

In conclusion, in the present study we have analyzed the blood transcriptome of 255 commercial Duroc pigs, reporting more than 23 million associations between 5,682,600 variants and 6,051 genes. We have characterized *cis*, *trans* and hotspot regulatory variants of the blood transcriptome across the pig genome and annotated the most notable ones associated with immunity. The annotation of the identified *cis-*regulatory variants remarked the importance of considering 3’UTR as regulatory regions. Additionally, we compared our results with previous studies on blood and other pig tissue transcriptomes identifying commonly regulatory variants. The colocalization studies of eQTL with QTLs associated with health traits revealed a list of potential causative mutations regulating immunocompetence in pigs. All these data contribute to expand our knowledge of porcine gene expression regulation and provide insights into the regulatory mechanisms that shape immunity-related phenotypes.

## Data Availability

The raw sequence data used in this study have been deposited in the SRA repository with the Bioproject accession code PRJNA1267984. Significant associations detected through eGWAS have been made publicly available in CORA.RDR repository (Doi: https://doi.org/10.34810/data2296).

## Supplementary material

Supplementary Figure 1 Pie charts representing the consequences of **(A)** all *cis*-associated variants; **(B)** *cis*-variants in coding regions.
