# Supplementary figures and images for: Unveiling regulatory variants in the blood transcriptome and their association with immunity traits in pigs
Source: Front Immunol. 2025 Jun 5;16:1582982. doi: 10.3389/fimmu.2025.1582982 (PMC12177557; doi:10.3389/fimmu.2025.1582982)

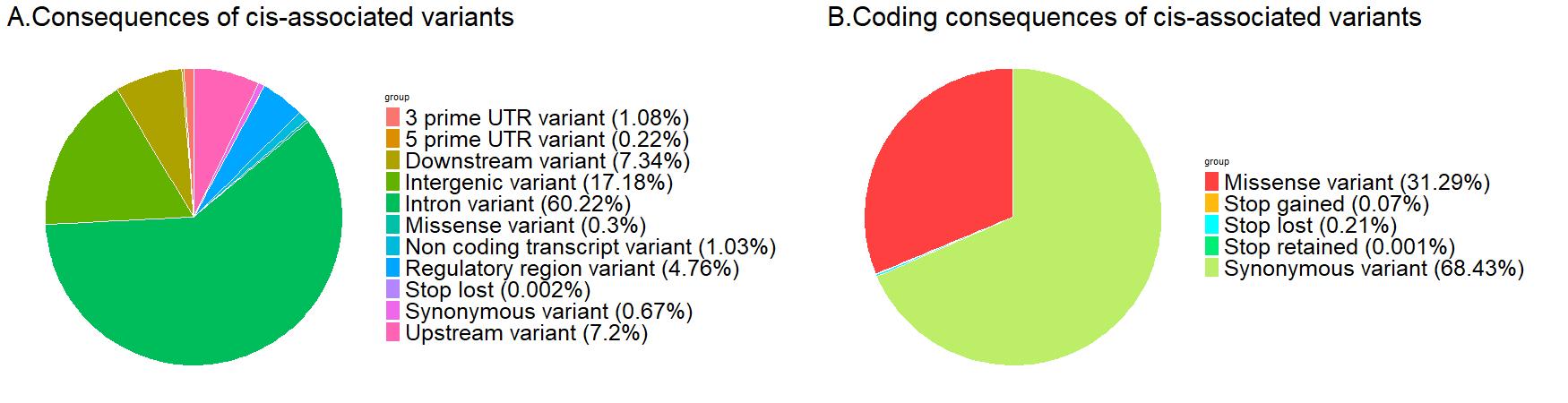

Supplement: Supplementary Figure 1 — Pie charts representing the consequences of (A) all cis-associated variants; (B) cis-variants in coding regions. [file Image1.tiff]
